# How does mindfulness affect media employees’ creative engagement? The chain-mediation model of institutional pressures and career adaptability

**DOI:** 10.3389/fpsyg.2025.1617956

**Published:** 2025-09-24

**Authors:** Meiying Wu, Yongxuan Gao

**Affiliations:** ^1^Lingnan Culture Digital Research Center, Dongguan City University, Dongguan, China; ^2^Faculty of Humanities and Social Sciences, City University of Macau, Macau, China

**Keywords:** institutional pressures, employee mindfulness, Chinese media, creative engagement, career adaptability, media psychology

## Abstract

**Background:**

Artificial intelligence is reshaping media production, forcing professionals to confront stringent institutional pressures and rising innovation demands. Although employee mindfulness is a critical psychological resource, its mechanism for fostering creative engagement under high constraints remains unclear.

**Objective:**

This study examines how employee mindfulness influences creative engagement through a chain-mediation model of institutional pressures and career adaptability among Chinese media professionals.

**Methods:**

Data from 804 media professionals were analyzed using hierarchical regression and PROCESS Macro (Model 6) to test direct and indirect effects, controlling for demographic variables.

**Results:**

(1) Employee mindfulness, institutional pressures, and career adaptability each have significant direct predictive effects on media professionals’ creative engagement; (2) Institutional pressures and career adaptability play a chain mediating role in the relationship between employee mindfulness and creative engagement, with this mediating effect involving three pathways: employee mindfulness → institutional pressures → creative engagement (indirect effect = 0.094, 95% CI = [0.060, 0.134], accounting for 25.54% of the total effect); employee mindfulness → career adaptability → creative engagement (indirect effect = 0.069, 95% CI = [0.043, 0.098], accounting for 18.75% of the total effect); employee mindfulness → institutional pressures → career adaptability → creative engagement (indirect effect = 0.042, 95% CI = [0.027, 0.060], accounting for 11.41% of the total effect).

**Conclusion:**

Mindfulness promotes creative engagement by reducing institutional pressures and enhancing career adaptability, revealing a “cognitive restructuring-capability enhancement” mechanism. Findings offer theoretical and practical insights for balancing compliance and innovation in media organizations.

## 1 Introduction

In recent years, artificial intelligence (AI) has been reshaping value orientation within the media production ecosystem. As AI continues to enhance the efficiency of information production (Li, 2022), algorithmic automation is increasingly undertaking fundamental content generation tasks (Dovbysh, 2020). Consequently, creativity has become the core competitiveness of media professionals (Zhou and Lee, 2024).

However, given the media industry’s unique role in shaping national ideology (Reynolds, 2019), content regulation tends to be more stringent (Yesil, 2014). This institutional environment requires media professionals worldwide to balance compliance with regulatory frameworks against the need to maintain professional autonomy and innovation (Reynolds, 2019). This dynamic reflects the “exploration – exploitation” paradox originally proposed by March (1991). For media professionals, exploration entails the pursuit of novelty and creative autonomy—such as innovating narrative forms and developing original content—while exploitation demands efficiency and adherence to standardized processes, including regulatory compliance, meeting deadlines, and reproducing reliable content. This paradox creates a fundamental tension for media professionals (Knight and Harvey, 2015), who are simultaneously “pulled” toward creative expression and “pushed” toward compliant output (Orhan and Scott, 2001). For instance, a journalist may be driven to investigate a critical story (exploration), yet feel compelled to avoid sensitive topics to uphold organizational legitimacy (exploitation), thereby intensifying self-censorship over content production (Arsan, 2013; Lee et al., 2023). Such continual negotiation presents not only an operational challenge but also a profound psychological strain: the processes of self-censorship and constant balancing deplete cognitive resources. It is within this tension that we position mindfulness as a critical resource to help media professionals navigate the paradox, alleviate its attendant stress, and foster creative engagement within constrained environments.

The Chinese media ecosystem is precisely a microcosm of the exploration-exploitation conflict. As of January 2025, the number of Internet users in China reached 1.108 billion, and the Internet penetration rate rose to 78.6% (The Ministry of Industry and Information Technology of the People’s Republic of China, 2025). The large-scale internet user base promotes content democratization while giving rise to strict platform governance mechanisms, reflecting the common phenomenon of the interplay between innovation and control on a global scale.

This study focuses on Chinese media professionals, defined as: (a) institutional media professionals, categorized according to the ISCO-08 standard as core-position personnel in licensed media organizations who engage in full-time content production (ILO, 2012); (b) those who systematically address three types of institutional pressures (coercive, mimetic, and normative pressures); (c) individuals directly producing works for public dissemination (excluding support roles).

Current research on media professionals’ creative investment largely focuses on organizational incentives, talent development, and competency enhancement (Kirchhoff, 2022; Malmelin and Virta, 2016). Little research has yet attempted to address the “exploration-exploitation” paradox experienced by media professionals from a micro-level psychological process perspective. This research aims to fill this gap from a psychological perspective.

Mindfulness, a positive psychological state, has received considerable scholarly interest in recent years. Defined as non-judgmental awareness of the present moment (Kabat-Zinn, 2005), it is considered a psychological resource that can effectively alleviate stress and enhance cognitive functioning (Hobfoll, 1989). Empirical studies have demonstrated its numerous positive effects in general workplace contexts. For example, mindfulness has been shown to improve employees’ self-regulation (Garofalo et al., 2020), enhance psychological resilience and emotional stability (Oh et al., 2022; Hulsheger et al., 2013), reduce work-related stress, and increase job satisfaction (Bolm et al., 2022). It also facilitates knowledge-sharing behaviors (Zhang et al., 2024) and is associated with positive psychological mechanisms underlying creativity (He, 2024). These findings provide a theoretical foundation for exploring how mindfulness influences creative investment among media professionals.

However, despite the widely recognized benefits of mindfulness, notable gaps remain in current research. On the one hand, most studies focus on its role in low-constraint environments, with little exploration of its mechanisms in high-institutional-pressure contexts like the media industry. On the other hand, there is still no clear conclusion regarding the chain of mechanisms through which mindfulness transforms external constraints into individual creative engagement. Although mindfulness has demonstrated positive effects in multiple domains, its application, and underlying mechanisms in specific high-stress occupational contexts, such as media practice, still await further investigation.

Against this backdrop, this study aims to deeply explore how mindfulness influences the creativity of media professionals. Specifically, we construct a theoretical model grounded in the complementary perspectives of Institutional Theory and the Conservation of Resources Theory (COR). Institutional Theory defines the external pressures constraining media professionals, while COR theory explains how these pressures deplete the cognitive resources necessary for creativity. We propose that mindfulness, as a resource, counters this by reducing perceived pressures and enhancing career adaptability, thereby facilitating creative engagement through a chain-mediation process. The research questions are as follows: (a) This study explores whether mindfulness has a positive impact on media professionals’ perception of institutional pressures, occupational adaptability, and creative investment. (b) This study investigates whether institutional pressures and occupational adaptability mediate the relationship between mindfulness and creative investment. (c) This study examines whether a chain mediation effect exists between mindfulness and creative investment, with institutional pressures and occupational adaptability serving as sequential mediators.

Theoretically, this study advances a chained mediation model of “institutional pressures → career adaptability → creative engagement,” elucidating the nuanced mechanisms through which employee mindfulness propels creative engagement under highly constrained conditions. In doing so, it extends the boundary conditions of mindfulness theory within organizational scholarship and furnishes a resource-conservation-based micro-psychological lens for resolving the exploration-exploitation paradox. Practically, the findings can help media professionals maintain creative competitiveness amid pressures from algorithmic substitution and provide policy implications for media regulatory authorities worldwide.

## 2 Literature review

### 2.1 Institutional theory

Institutional Theory originated in 20th-century sociological and organizational research, with its core focusing on how organizational behavior is influenced by external institutional environments (Scott, 2008). Meyer and Rowan (1977) introduced the concept of “institutional environment,” arguing that organizational structures often embody institutional myths. Organizations not only pursue technical efficiency but, more importantly, seek legitimacy, a phenomenon leading to the separation between formal structures and actual operations, which is known as “decoupling” (Haack and Schoeneborn, 2015; Jastram and Foersterling, 2024).

Scott (1995) expanded Institutional Theory, emphasizing that the institutional environment is composed of three core pillars, namely regulative, normative, and cognitive, which collectively shape organizational structures and behaviors. This theory explains not only why organizations adopt seemingly inefficient structures but also how they maintain legitimacy and viability in dynamic institutional contexts (Scott, 2008). Paul and Powell (2010) identified three types of isomorphic pressures stemming from institutional environments that act on organizations: coercive, mimetic, and normative pressures.

Coercive pressure arises from formal institutional mandates, such as laws, regulations, and policies. A typical example in the media industry is content censorship stipulated under cybersecurity laws (Feng et al., 2023). Mimetic pressure refers to the tendency to imitate industry benchmarks or competitors. This can be observed in content convergence driven by the growing trend toward algorithmic transparency on social media platforms (Etta, 2024), a phenomenon often associated with bandwagon effects. Normative pressure, on the other hand, stems from professionalization and is rooted in informal norms embedded within social culture and professional ethics, such as the ethical guidelines observed by journalists.

In recent years, the application of Institutional Theory in media studies has continuously expanded, particularly demonstrating unique value in explaining how media professionals navigate the multiple pressures of policy regulation, market competition, and cultural expectations (Reynolds, 2019; Yesil, 2014). In the media industry, institutional pressures exhibit characteristics of multi-source and dynamism, encompassing both explicit policies and regulations and implicit cultural expectations. These pressures influence individual behavior through cognitive embedding mechanisms: explicit policies are enforced via clear reward-punishment systems, while implicit expectations subtly shape cognition through channels such as public opinion and peer evaluation.

This pressure causes media professionals to find themselves caught in a contradiction between innovative demands and compliance requirements, which is a concrete manifestation of the “decoupling” phenomenon (Jastram and Foersterling, 2024; Meyer and Rowan, 1977). In this study, Institutional Theory provides a crucial framework for understanding how media professionals respond to institutional pressures. Through this lens, we can better analyze how practitioners make decisions amid multiple constraints and limited psychological resources, as well as the broader implications of these choices for the development of the media industry.

### 2.2 Conservation of resources theory

The Conservation of Resources (COR) theory, proposed by Hobfoll (1989), has been widely applied to study the impact of external stress on organizations (Lai et al., 2006). This theory emphasizes that individuals have a strong motivation to acquire, protect, and maintain valuable resources, including objects, personal characteristics, conditions, and energies. Later, Halbesleben et al. (2014) revised the definition of resources, stating that a resource is anything an individual perceives as helpful in achieving their goals.

Conservation of Resources theory is particularly important for understanding stress in work organizations. Its core premise is that stress arises when individuals face the threat of resource loss, experience actual resource loss, or fail to obtain expected resource gains after investing resources. To avoid resource loss or achieve resource accumulation, individuals adopt various coping strategies (Hobfoll et al., 2018).

The COR theory, widely applied in organizational stress research, provides a powerful framework for understanding the relationship between employee stress and work performance. Although this theory has been applied to numerous professional contexts, its application to Chinese media professionals remains limited. As one of the key stressors in the media industry, the impact of institutional pressure on the work performance of Chinese media employees has not been fully explored.

According to COR theory, employee mindfulness can be viewed as an important personal resource, a stable personal trait that helps maintain a state of present-focused awareness (Glomb et al., 2011). This trait helps them identify and accept negative emotions at work, allowing them to cope with job challenges with a calmer and more stable mindset (Schultz et al., 2015). Perry et al. (2011) argued within COR theory that “key resources” are those that help individuals manage other resources, and employee mindfulness aligns precisely with this definition.

In summary, the COR theory provides a theoretical framework for examining the mechanisms linking employee mindfulness, institutional pressures, career adaptability, and creative engagement. From this theoretical perspective, we can delve into how employees leverage mindfulness, a key personal resource, to cope with institutional pressures, thereby influencing their career adaptability and creative engagement. Thus, it offers a critical theoretical lens for unraveling how media employees’ performance is shaped by the dynamic interplay between stress and resources.

### 2.3 An integrated framework of institutional and conservation of resources theories

While Institutional Theory and Conservation of Resources Theory originate from distinct scholarly traditions, they offer complementary perspectives for understanding the challenges media professionals face. Institutional Theory provides a macro-level lens, elucidating the origin and nature of external pressures—such as coercive, mimetic, and normative forces—that constrain individual action. In contrast, COR Theory offers a micro-level, psychological lens, explaining the internal mechanism through which these institutional pressures function as “resource depletion demands.” Such demands threaten an individual’s cognitive and emotional resources, thereby inhibiting resource-intensive activities like creative engagement.

The intersection of these two theories lies in conceptualizing mindfulness as a critical personal resource (Hobfoll et al., 2018). We propose that mindfulness, by enhancing present-moment awareness and non-judgmental acceptance, enables individuals to cognitively reframe institutional pressures (mitigating their resource-depleting nature) and to bolster their resource reservoirs, which can then be invested into developing career adaptability and, ultimately, creative engagement. Thus, an integrated framework allows us to posit that mindfulness influences creative outcomes through its role in helping individuals manage the resource threats posed by the institutional environment.

### 2.4 Employee mindfulness and creative engagement

Employees’ creativity is crucial for organizational development, as it is demonstrated not only through solving problems in novel and efficient ways but also through generating useful ideas tailored to specific needs such as processes, services, and methods (Gilson, 2008). Additionally, Saridakis et al. (2017) argues that enhancing employees’ knowledge and skills, motivation, and capacity for engagement can boost creativity and innovative behavior. In complex media work environments, employees often need to identify problems independently and devise solutions, with their creative ideas needing to be relevant to the media organization’s tasks and compliant with institutional regulations. Overall, problem identification, information search, and creative idea generation are key components of creative activities in the workplace (Reiter-Palmon and Illies, 2004).

We argue that employee mindfulness directly enhances creative engagement for several key reasons. First, mindfulness helps individuals reduce automatic thinking, practice acceptance, and maintain a non-judgmental attitude (Ovington et al., 2018). Individuals with high mindfulness can respond to situations in a non-automatic manner, and “non-automaticity” is considered a critical factor in fostering individual creative activities (Dane and Rockmann, 2020; Kudesia, 2015). As such, employees’ mindfulness allows them to better assess their current creative potential, facilitate the absorption and integration of ideas, and ultimately generate creative engagement in their work (Veiskarami and Yousefvand, 2018). Second, media work is often accompanied by stress and anxiety, negative factors that tend to inhibit employees’ creative engagement (Anasori et al., 2023). Mindfulness helps individuals enter a cognitively creative mental state at work; that is, mindful individuals can think more clearly and overcome dominant but uncreative responses (Op den Kamp et al., 2023).

Additionally, research shows that mindfulness enables individuals to better understand others’ perspectives and feelings, enhancing interpersonal skills and internal team communication (Adamu et al., 2023). Active, autonomous, and flexible organizational communication is conducive to stimulating employees’ innovative activities (Fraihat et al., 2023). Meanwhile, from the perspective of Conservation of Resources (COR) theory, individuals tend to invest personal resources to increase the likelihood of acquiring other resources. Thus, mindfulness, as an important individual resource, may be utilized by employees to generate additional new resources or develop new skills. In summary, these arguments suggest that there is likely a positive correlation between employee mindfulness and creative engagement.

Therefore, we propose the following hypothesis:

H1: Employee mindfulness has a positive effect on creative engagement.

### 2.5 The mediating role of career adaptability

Career adaptability is a psychosocial construct that focuses on individuals’ adaptive resources and capabilities within the occupational domain (Savickas, 1997) defined it as the psychological resources that enable individuals to cope with current and anticipated tasks, transitions, and traumas, which in turn influence their level of social integration. This construct comprises four dimensions: concern, control, curiosity, and confidence. “Concern” refers to employees’ ability to proactively envision their future and prepare for career development; “control” represents their clarity about career aspirations and the agency to make deliberate, responsible decisions; “curiosity” reflects their proactivity in exploring future career opportunities and potential possibilities; and “confidence” refers to employees’ self-efficacy in performing activities necessary to achieve career goals (Savickas and Porfeli, 2012).

Existing research suggests that employee mindfulness may enhance career adaptability. Studies indicate that mindfulness is associated with several positive outcomes conducive to career development, such as reduced work stress, lower job burnout, and decreased turnover intentions (Qi et al., 2024), as well as improved well-being and job performance (Christodoulou et al., 2025). Additionally, mindfulness helps strengthen employees’ self-awareness, self-regulation, and self-transcendence (Lemmon et al., 2025), enabling employees to make decisions that are better aligned with their personal and situational needs. This supports meaningful adaptation in the workplace.

For individuals, engaging in creativity-related activities requires relaxation or flow states (to enhance focus), a willingness to take risks (without fear of judgment), as well as curiosity and openness to new experiences (to reduce self-consciousness, i.e., the fear of negative social evaluation) (Prabhu et al., 2008), elements that closely align with the four dimensions of career adaptability: concern, control, curiosity, and confidence. Meanwhile, career adaptability is likely importantly linked to employees’ creative engagement, as individuals with strong career adaptability are better at proactively exploring career choices and ways of working, thereby improving their work capabilities and performance (Federici et al., 2021). Additionally, research by Zywiolek et al. (2022) indicates that employee adaptability mediates the relationship between transformational leadership and creativity, suggesting a connection between adaptive capabilities and creativity.

Therefore, we propose the following hypotheses:

H2: Employee mindfulness has a positive effect on career adaptability.

H3: Career adaptability has a positive effect on creative engagement.

H4: Career adaptability mediates the relationship between employee mindfulness and creative engagement.

### 2.6 The mediating role of institutional pressures

Institutional pressures refer to the stress that individuals or organizations experience due to external institutional environments. These pressures originate from formal institutions (e.g., laws, regulations, policies, industry standards) and informal norms (e.g., cultural cognitions, social values) (Paul and Powell, 2010; Scott, 2008). Vanalle et al. (2017) classified institutional pressures into three types: coercive pressure, mimetic pressure, and normative pressure. According to Conservation of Resources (COR) theory (Hobfoll, 1989), individuals adopt various coping strategies to protect resources when facing threats of resource loss. As a key personal resource (Hobfoll et al., 2018), mindfulness can reduce the perceived threat of institutional pressures by enhancing individuals’ ability to cognitively reconstruct stressors (Dahl et al., 2015).

Existing research indicates that individuals with high mindfulness are better at identifying controllable aspects within institutional pressures, such as policy compliance requirements, thereby avoiding excessive resource depletion and promoting sustainable resource management (Ndubisi and Al-Shuridah, 2019). By adopting a non-judgmental attitude, mindfulness helps individuals reduce resistance to coercive pressure and minimize the consumption of psychological resources (Lee et al., 2024), enabling employees to cope with work challenges with a calmer and more stable mindset (Orosz et al., 2024). Therefore, employee mindfulness mitigates the negative impacts of institutional pressure.

In the media industry, institutional pressures mainly take the form of content censorship mechanisms, professional ethical standards, and market competition demands (Reynolds, 2019; Yesil, 2014). As Reynolds (2019) observes, media professionals must navigate such institutional pressures during news production, especially in balancing ideological expectations with journalistic professionalism when creating content (Zollmann, 2017). This balancing act essentially constitutes a cognitive embedding process through which individuals adapt to institutional pressures. Self-censorship, as an expression of cognitive embedding, occurs when employees adjust their behaviors to meet the organization’s legitimacy needs, a form of adaptation that imposes additional psychological and physical resource costs on employees. To maintain their own resource balance (Hobfoll, 1989), individuals may reduce investments in creative resources. Additionally, market competition pressures may lead employees to overly focus on short-term performance, neglecting long-term creative engagement (Reynolds, 2019). Based on this, we infer that institutional pressures have a negative impact on creative engagement.

Therefore, we propose the following hypotheses:

H5: Employee mindfulness has a negative effect on institutional pressures.

H6: Institutional pressures have a negative effect on creative engagement.

H7: Institutional pressures mediate the relationship between employee mindfulness and creative engagement.

### 2.7 The chain-mediating role of institutional pressures and career adaptability

The essence of institutional pressures lies in the constraints imposed on individual behavior by organizational and industry norms (Scott, 2008). According to institutional theory (Paul and Powell, 2010), individuals must reconcile different logics to achieve professional legitimacy, which generates stress at the individual level (Friedland, 2012). Institutional pressures challenge individuals’ occupational identity, and they reconcile their occupational identity with behavioral performance through career adaptability (Ashforth and Schinoff, 2016). As an ability to proactively adjust skills, behaviors, and attitudes to adapt to changes in the occupational environment (Savickas, 1997), career adaptability helps individuals reconstruct meaning amid identity conflicts, thereby reducing cognitive dissonance. Thus, career adaptability essentially reflects a resource management capability, allowing individuals to optimize the allocation and regeneration of resources under constraints such as institutional pressures.

According to Conservation of Resources theory (Hobfoll, 1989), institutional pressures compel individuals to invest substantial resources to comply with external requirements, resulting in diminished resource reserves. This depletion may activate a resource conservation strategy, leading individuals to curtail further investment in order to safeguard basic functioning. Existing research by Rudolph et al. (2017) has shown that career adaptability is significantly inhibited by work stress, and institutional pressures, due to their coercive and uncontrollable nature, have a more pronounced negative impact on adaptability, especially in highly institutionalized environments. In such contexts, pressure originates not only from task demands but also from the rigid constraints of external regulations (Bowling, 2024), which can erode career adaptability more severely than general job stress. We therefore propose that institutional pressures undermine career adaptability through a resource depletion mechanism, while career adaptability itself may serve as a buffering resource that helps mitigate the negative influence of these pressures.

Therefore, we propose the following hypothesis:

H8: Institutional pressures have a negative effect on career adaptability.

H9: Institutional pressures and career adaptability together exhibit a chain mediating effect in the relationship between employee mindfulness and creative engagement.

**FIGURE 1:**
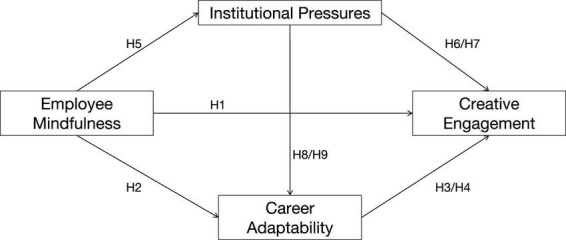
The hypothetical structure model.

Overall, this study constructs a chain mediating model involving employee mindfulness, institutional pressures, career adaptability, and creative engagement (Figure 1).

## 3 Research participants and methods

### 3.1 Participants and sampling

This study employed convenience sampling to recruit research participants. The target population comprised institutional media practitioners from diverse Chinese media organizations, including traditional media (e.g., newspapers, magazines, radio, and television stations) and new media platforms (e.g., online news portals, social media content providers, and video-sharing platforms). Consistent with distinctions in the literature (ILO, 2012), we focused solely on organization-affiliated professionals, excluding independents and freelancers to homogenize the sample regarding institutional pressures.

Questionnaires were distributed through dual channels: online and offline. Online dissemination involved professional media-related platforms (e.g., Zhihu, Douban), industry-specific social media groups, and email invitations to media professionals. In all online recruitment advertisements, we explicitly stated that the survey was targeted at full-time employees directly involved in content production within media organizations to ensure participants met our sampling criteria. Offline, we partnered with media organizations to distribute questionnaires directly to attending practitioners at media conferences, seminars, and training events. Through our partner organizations’ management, questionnaires were targeted to employees in content production departments. Prior to data collection, participants received standardized instructions on survey procedures and objectives and provided informed consent via signed forms. The study was approved by the Ethics Committee of Dongguan City University.

**FIGURE 2:**
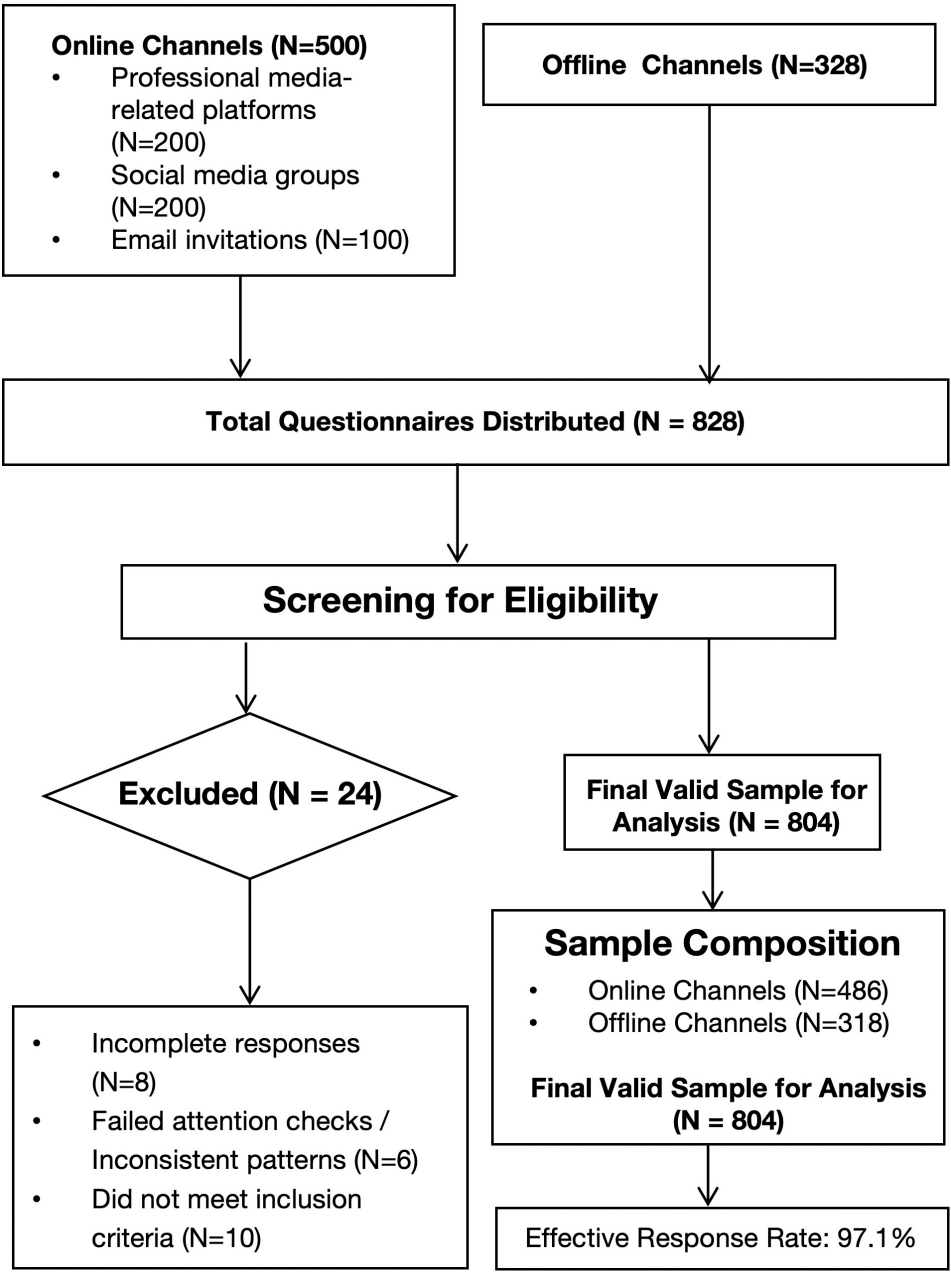
Participant flow diagram. Note: This diagram illustrates the process of participant recruitment and data screening, culminating in the final valid sample size for data analysis (*N* = 804).

A total of 828 questionnaires were distributed (500 online and 328 offline). After screening, 804 valid questionnaires were finally obtained. Invalid ones were removed for reasons like incomplete answers, inconsistent response patterns, or not meeting sample criteria (such as non-media professionals filling out the survey by mistake), giving an effective response rate of 97.1%. The participant flow is summarized in Figure 2.

### 3.2 Research tools

For this study, responses were rated using a five-point Likert scale ranging from one (strongly disagree) to five (strongly agree).

#### 3.2.1 Employee mindfulness

Employee Mindfulness was evaluated using the Mindful Attention and Awareness Scale (MAAS) developed by Brown and Ryan (2003). A sample item from the scale is: “I find it difficult to stay focused on what’s happening in the present”. The scale exhibited excellent reliability (Cronbach’s α = 0.954) and good validity (χ^2^/df = 1.272, GFI = 0.981, AGFI = 0.974, IFI = 0.997, TLI = 0.996, CFI = 0.997, RMSEA = 0.018).

#### 3.2.2 Institutional pressures

Institutional pressures were measured using the 9-item scale developed by Bharati et al. (2014), which assesses three conceptually distinct dimensions:

Coercive pressures: Formal mandates from external institutions (e.g., laws, stakeholder requirements); sample item: “We spend considerable time on meetings and telephone conversations with our important customers.”

Normative pressures: Pressures stemming from professionalization, shared social norms, or occupational ethics; sample item: “We share the same vision of the industry as our competitors.”

Mimetic pressures: Tendency to imitate competitors/industry benchmarks to reduce uncertainty; sample item: “Our main competitors are adopting new technologies.”

The Cronbach’s alpha was 0.862. The fitting index of confirmatory analysis of the scale: χ^2^/df = 1.944, GFI = 0.987, AGFI = 0.975, IFI = 0.993, TLI = 0.989, CFI = 0.993, RMSEA = 0.034, indicating that the scale has good reliability and validity.

#### 3.2.3 Career adaptability

Career adaptability was assessed using the 24-item Career Adapt-Abilities Scale (CAAS) developed by Savickas and Porfeli (2012), which comprises four dimensions:

Concern: Planning for one’s future career; sample item: “Concerned about my career.”

Control: Taking responsibility for shaping one’s career; sample item: “Counting on myself.”

Curiosity: Exploring future career possibilities; sample item: “Exploring my surroundings.”

Confidence: Believing in one’s ability to achieve career goals; sample item: “Performing tasks efficiently.”

The scale showed high reliability (Cronbach’s α = 0.938) and good validity (χ^2^/df = 1.706, GFI = 0.954, AGFI = 0.943, IFI = 0.984, TLI = 0.982, CFI = 0.984, RMSEA = 0.030).

#### 3.2.4 Creative engagement

The 13-item Scale developed by Contreras et al. (2017) and Li and Sandino (2018) to measure creative engagement. The sample item is: “During my workday, I strive to solve problems using new methodologies” The scale demonstrated high reliability (Cronbach’s α = 0.947) and good validity (χ^2^/df = 1.532, GFI = 0.981, AGFI = 0.973, IFI = 0.995, TLI = 0.994, CFI = 0.995, RMSEA = 0.026).

### 3.3 Data analysis

Statistical analyzes were conducted using SPSS 26.0 and PROCESS 4.2. First, Harman’s single-factor test was employed to assess common method bias. Subsequent analyzes included descriptive statistics and evaluation of each scale’s reliability via Cronbach’s alpha. Confirmatory factor analysis (CFA) was performed using Amos 26.0 to validate the factor structure of the four questionnaires. Pearson correlation coefficients were computed to examine bivariate relationships between variables. Finally, PROCESS macro (Model 6) was utilized with 5,000 bootstrap samples to generate bias-corrected 95% confidence intervals to investigate the chain mediating relationships among employee mindfulness, institutional pressures, career adaptability, and creative engagement, while controlling for gender, age, education level, work experience, and monthly income.

## 4 Research results

### 4.1 Common method bias test

Collecting all questionnaire data through self-reported responses from a single source may introduce common method bias, which is typically assessed using Harman’s single-factor test (Podsakoff et al., 2003). When the variance explained by the first factor in Harman’s test exceeds 40%, significant common method bias is considered present. In this study, the first factor accounted for 30.199% of the total variance, below the established threshold, indicating that no serious common method bias affected the survey results.

### 4.2 Descriptive statistics and correlation analysis of each variable

The descriptive statistics of each variable in this study are presented in Table 1, and the correlation analysis is shown in Table 2. The results indicate that the correlations among all variables reach a significant level, as follows: There is a significant negative correlation between employees’ mindfulness and institutional pressures (*r* = −0.452, *p* < 0.001). Employees’ mindfulness is significantly positively correlated with both career adaptability and creative engagement (*r* = 0.458, *p* < 0.001; *r* = 0.370, *p* < 0.001). Institutional pressures are negatively correlated with career adaptability (*r* = −0.513, *p* < 0.001) and creative engagement (*r* = −0.405, *p* < 0.001). Additionally, career adaptability is positively correlated with creative engagement (*r* = 0.425, *p* < 0.001). It should be noted that a higher score on the institutional pressures scale indicates that employees feel stronger constraints on their behavior from organizational or industry norms. Therefore, institutional pressures are negatively correlated with both career adaptability and creative engagement. The correlations among the variables provide a basis for the subsequent hypothesis testing.

**TABLE 1:** Demographic characteristics of participants (*N* = 804).

Characteristics	Frequency	Percent (%)
**Gender**		
Male	399	49.63%
Female	405	50.37%
**Age**		
18–25	99	12.31%
26–30	264	32.84%
31–40	221	27.49%
41–50	138	17.16%
51–60	55	6.84%
Over 60	27	3.36%
**Education**		
Secondary school graduate	131	16.29%
Junior college diploma	242	30.1%
Bachelor’s degree	337	41.92%
Master’s degree and above	94	11.69%
**Work experience**		
Less than 3 years	202	25.12%
3–5 years	193	24%
5–10 years	146	18.16%
Over 10 years	263	32.71%
**Monthly income**		
3,000–5,000 rmb	288	35.82%
5,001–10,000 rmb	422	52.49%
10,001–15,000 rmb	62	7.71%
More than 15,000 rmb	32	3.98%

Note. All participants were confirmed to be institutional media professionals (i.e., full-time content production personnel affiliated with licensed media organizations).

**TABLE 2:** Descriptive statistics and correlation matrix of each variable (*N* = 804).

Variable	M	SD	1	2	3	4
EM	3.352	0.892	**-**			
IP	2.621	0.813	−0.452***	**-**		
CA	3.432	0.747	0.458***	−0.513***	**-**	
CE	3.331	0.895	0.370***	−0.405***	0.425***	**-**

Notes. *N* = 804. EM: employee mindfulness. IP: institutional pressures. CA: career adaptability. CE: creative engagement.

***p < 0.001.

### 4.3 Examination of the mediating effects

We developed a multiple regression model with employees’ mindfulness, institutional pressures, and career adaptability as independent variables, and creative engagement as the dependent variable. The variance inflation factor (VIF) values were all less than 5, indicating no multicollinearity among the predictor variables. To further examine the relationships among employees’ mindfulness, institutional pressures, career adaptability, and creative engagement, we performed hierarchical regression analysis using Model 6 of the SPSS macro PROCESS developed by Hayes. Taking employees’ mindfulness as the independent variable, creative engagement as the dependent variable, and gender, age, education level, work experience, and monthly income as control variables—while considering structural relationships in the model (such as potential paths involving institutional pressures and career adaptability), we conducted regression analysis, with results presented in Table 3.

**TABLE 3:** Regression analysis of the research variables.

Variable	CE (before controlling for variables)			IP			CA			CE (after controlling for variables)		
	β	*t*	SE	β	*t*	SE	β	*t*	SE	β	*t*	SE
Gender	−0.074	−2.267	0.059	−0.016	−0.511	0.051	0.014	0.476	0.043	−0.082	−2.695	0.055
Age	0.019	0.261	0.051	−0.080	−1.174	0.045	−0.047	−0.739	0.038	0.006	0.086	0.048
Education	0.078	1.839	0.042	−0.081	−1.995	0.037	−0.017	−0.447	0.031	0.057	1.451	0.039
Work experience	0.038	0.576	0.050	−0.035	−0.556	0.044	0.075	1.282	0.037	0.009	0.148	0.047
Monthly income	0.028	0.795	0.043	0.067	1.962	0.037	0.039	1.227	0.032	0.039	1.172	0.040
EM	0.367	11.222***	0.033	−0.452	−14.385***	0.029	0.283	8.701***	0.027	0.162	4.516***	0.036
IP							−0.385	−11.770***	0.030	−0.208	−5.566***	0.041
CA										0.243	6.515***	0.045
*R* ^2^	0.149			0.215			0.332			0.260		
Adjusted *R*^2^	0.143			0.209			0.327			0.253		
*F*	23.331***			36.334***			56.632***			34.941***		

Notes. *N* = 804. EM: employee mindfulness. IP: institutional pressures. CA: career adaptability. CE: creative engagement.

***p < 0.001.

Results showed that employees’ mindfulness not only positively predicted career adaptability (β = 0.283, *t* = 8.701, *p* < 0.001) but also negatively predicted institutional pressures (β = −0.452, *t* = −14.385, *p* < 0.001). After controlling for demographic variables, institutional pressures had a significant negative effect on creative engagement (β = −0.208, *t* = −5.566, *p* < 0.001), while career adaptability had a significant positive effect on creative engagement (β = 0.243, *t* = 6.515, *p* < 0.001). The direct effect of employees’ mindfulness on creative engagement remained significant (β = 0.162, *t* = 4.516, *p* < 0.001), indicating that employees’ mindfulness can directly promote creative engagement and may also indirectly influence it through the mediating effects of institutional pressures and career adaptability.

Subsequently, the study used the Bootstrap method (with the number of resampling times set at 5,000 and the confidence interval set at 95%) to test the chain mediating model. Results showed that the total effect of employee mindfulness on creative engagement was 0.368 [95% CI = (0.304, 0.433)], and the direct effect was 0.163 [95% CI = (0.092, 0.233)], indicating a significant direct effect of employees’ mindfulness on creative engagement, supporting Hypothesis H1. The effects of the three mediating paths were as follows:

Path 1 (employee mindfulness → institutional pressures → creative engagement): The indirect effect was 0.094 (95% CI = [0.060, 0.134]), accounting for 25.54% of the total effect. The confidence interval did not include 0, indicating that institutional pressure played a significant mediating role in the relationship between employee mindfulness and creative engagement, supporting Hypothesis H7.

Path 2 (employee mindfulness → career adaptability → creative engagement): The indirect effect was 0.069 (95% CI = [0.043, 0.098]), accounting for 18.75% of the total effect. The confidence interval did not include 0, indicating a significant mediating effect of career adaptability, supporting Hypothesis H4.

Path 3 (employee mindfulness → institutional pressures → career adaptability → creative engagement): The indirect effect was 0.042 [95% CI = (0.027, 0.060)], accounting for 11.41% of the total effect. The confidence interval did not include 0, confirming that institutional pressures and career adaptability formed a chain mediating effect. This finding supports H9, which posits that the two variables exhibit a chain mediating effect in the process by which employee mindfulness influences creative engagement (Table 4).

**TABLE 4:** Mediation effect analysis of institutional pressures and career adaptability.

		Effect	Boot SE	Boot LLCI	Boot ULCI	The ratio of indirect to total effect
Total effect		0.368	0.033	0.304	0.433	
Direct effect		0.163	0.036	0.092	0.233	
Indirect effect	Total indirect effect	0.206	0.022	0.163	0.250	56.00%
	Indirect effect 1	0.094	0.019	0.060	0.134	25.54%
	Indirect effect 2	0.069	0.014	0.043	0.098	18.75%
	Indirect effect 3	0.042	0.008	0.027	0.060	11.41%
	Compare 1	0.025	0.027	−0.027	0.079	
	Compare 2	0.052	0.022	0.009	0.095	
	Compare 3	0.027	0.012	0.003	0.053	

Boot SE and Boot LLCI and Boot ULCI, respectively, refer to the standard error of effect and lower and upper limits of 95% confidence interval estimated by the Bootstrap deviation correction method. Indirect effect 1: Employee Mindfulness → Institutional Pressures → Creative Engagement; Indirect effect 2: Employee Mindfulness → Career Adaptability → Creative Engagement; Indirect effect 3: Employee Mindfulness → Institutional Pressures → Career Adaptability → Creative Engagement.

**FIGURE 3:**
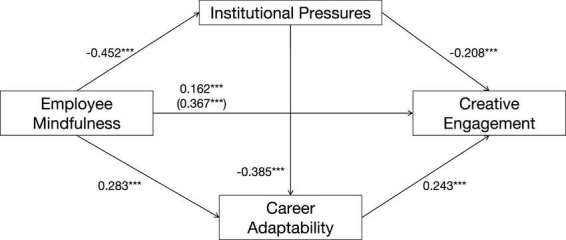
Chain mediation model of institutional pressures and career adaptability between employee mindfulness and creative engagement. (***p < 0.001).

Among the three indirect paths, Path 1 had the strongest effect (β = 0.094), accounting for 25.5% of the total effect. This suggests that mindfulness enhances creative engagement primarily by alleviating employees’ perceived institutional pressures. This finding is consistent with Conservation of Resources theory, which posits that conserving cognitive and emotional resources by reducing the threat of resource loss (e.g., from institutional pressures) frees up resources for more resource-intensive activities like creative work. In comparison, Path 2 and Path 3 exhibited smaller effects, though both remained statistically significant (Figure 3).

## 5 Conclusions and discussion

Grounded in an integrated framework of Conservation of Resources and institutional theory, this study reveals the mechanism through which mindfulness influences creative engagement among media practitioners in high-constraint environments. Empirical results first demonstrate the buffering effect of mindfulness as a psychological resource against institutional pressures, echoing Shapiro et al. (2005) classic discussion on the stress-buffering mechanism of mindfulness. Notably, this study expands the application boundaries of this mechanism through the lens of institutional theory: mindfulness not only plays a protective role via emotional regulation (Kabat-Zinn, 2005) but also transforms institutional pressures from “rigid restrictions” into “negotiable frameworks” by reconstructing individuals’ cognitive schemas of institutional constraints (Reynolds, 2019). This finding provides new evidence for understanding the cognitive restructuring function of mindfulness in organizational settings.

Furthermore, the study first confirms the chain mediating path between institutional pressures and career adaptability. This finding holds dual theoretical value: (1) From the Conservation of Resources perspective, it reveals the dynamic process by which mindfulness protects creative resources through a continuous mechanism of “cognitive restructuring-stress buffering-resource gain” (Hobfoll, 1989); (2) From the institutional theory perspective, it validates the key role of career adaptability as a micro-level agency factor (Haenggli and Hirschi, 2020) in breaking through meso-level institutional constraints. Notably, the impact of mindfulness on creative engagement can be realized through the chain path where institutional pressure acts on career adaptability, which complements the explanatory pathway of the career development perspective for Halbesleben et al. (2014) classic model of “stress-resource depletion.”

Additionally, this study further reveals three key mechanisms. (1) Institutional pressures directly inhibit creative engagement and indirectly suppress it by weakening career adaptability. This aligns with (Scott, 1995) discussion on the multidimensional effects of institutional pressures, indicating that institutional pressures not only directly restrict innovative behavior but also indirectly inhibit creative engagement by affecting career adaptability. (2) Career adaptability plays a significant independent mediating role between mindfulness and creative engagement. This finding expands (Savickas, 1997)’s career construction theory, suggesting that career adaptability is not just a survival skill for coping with environmental changes but a key transitional mechanism driving innovative behavior, playing an important role in how mindfulness influences creative engagement. (3) The significant chain mediating effect in this study shows that mindfulness generates synergistic effects through a continuous process of “cognitive restructuring → capability enhancement.” This cascading effect model provides a new analytical framework for understanding the complex pathways through which psychological traits influence innovative behavior, revealing a deeper mechanism by which mindfulness affects creative engagement.

## 6 Theoretical contributions

This study breaks through the organizational-level analysis of traditional institutional theory by revealing the micro-level consumption mechanism through which institutional pressures depletes individual resources. It thus offers a cross-level perspective on the classic interplay between “institutional constraints and individual initiative” (Friedland, 2012). Traditional institutional theory mainly focuses on how the macro-level institutional environment affects organizational behavior (Scott, 2008), but rarely explores the specific mechanism of institutional pressures at the individual level and its impact on individual initiative. By introducing mindfulness as a psychological resource, this study reveals how institutional pressures indirectly inhibit creative engagement by consuming resources. It also further clarifies how individuals can adjust their psychological states through mindfulness to mitigate the negative impact of institutional pressures. This finding not only expands the research boundary of institutional theory but also provides micro-mechanism support for understanding the dynamic relationship between institutional constraints and individual initiative.

Existing research on mindfulness has predominantly focused on low-pressure environments, examining its positive effects on mental health, emotional regulation, and innovation (Brown and Ryan, 2003; Kabat-Zinn, 2005). These studies often assume that stress remains manageable, allowing individuals to fully mobilize psychological resources to facilitate innovation. In contrast, this study places mindfulness in a high-constraint environment, indicating that mindfulness can not only function in low-pressure environments but also promote innovative behavior through dual paths (pressure relief and adaptability enhancement) in high-constraint situations. This result shows that mindfulness, as a psychological resource, can help individuals achieve “compliant innovation” (Paul and Powell, 2010) in high-constraint environments. That is, while complying with institutional constraints, individuals can explore innovative solutions by flexibly interpreting rules and optimizing resource management. Data analysis shows that mindfulness significantly reduces the negative impact of institutional pressures on career adaptability, thus making it possible for individuals to maintain creative engagement under constraints.

Additionally, this study offers a new perspective on exploration-exploitation paradox research (March, 1991) from a resource management angle. Paradox theory emphasizes how organizations and individuals can achieve the dual goals by integrating resources and maintaining dynamic balance when facing conflicting demands (Lewis, 2000). In a high-constraint environment, individuals often face the tension between “institutional constraints” and “innovation needs”. Traditional research usually regards this tension as a conflict to be avoided (such as by reducing constraints or lowering innovation risks). However, through the role of mindfulness, this study reveals how media professionals can achieve “compliant innovation” under institutional constraints. That is, while complying with institutional norms, they can meet innovation needs through creative behavior. This finding not only provides new empirical support for the exploitation-exploration paradox theory but also offers important practical implications for organizational managers: in high-constraint environments, they should focus on cultivating employees’ psychological resources (such as mindfulness) and dynamic capabilities (such as career adaptability) to effectively manage conflicting demands.

## 7 Practical implications

In terms of organizational interventions, the study highlights the important impact of mindfulness on creative engagement among media professionals. We recommend that media institutions design integrated “Mindfulness-Career Adaptability” training modules. This could begin with structured mindfulness programs (e.g., the 8-week Mindfulness-Based Stress Reduction protocol) to buffer the perception of institutional pressures, followed by skills-training workshops to enhance the four dimensions of career adaptability (Ma et al., 2024). For instance, workshops could use scenario-based simulations of common regulatory dilemmas to practice flexible and creative problem-solving. This approach can enhance psychological resilience (Oh et al., 2022), and help them transform the challenges brought by institutional constraints into opportunities for innovation, thereby increasing creative engagement (He, 2024).

On the institutional policy front, organizations should move beyond purely restrictive measures and design “supportive accountability” frameworks that balance rigidity with flexibility. Concretely, this involves:

(1) Creating formal “innovation grace periods”: This policy would shield pilot projects from short-term performance evaluations, insulating employees from the immediate perceived institutional pressures that our study identifies as a key barrier to creativity. By allowing a temporary space for exploration without the fear of failure, organizations can directly mitigate the hindering stressor measured in our research.

(2) Establishing clear “trial-and-error spaces”: This involves defining specific projects or channels where experimentation is explicitly encouraged and lessons from outcomes are shared as learning opportunities rather than punished. This approach reduces the punitive risks associated with innovation under constraint, thereby conserving employees’ cognitive resources for creative engagement rather than defensive compliance.

Additionally, when formulating institutional policies, organizations should focus on the rational allocation of resources to minimize the direct effects of institutional pressures on employees’ psychology and behavior. Policymakers must ensure that employees receive adequate resource support in high-pressure environments. For example, media organizations can offer flexible work arrangements to alleviate resource shortages caused by institutional constraints, thereby enhancing employees’ career adaptability and capacity for creative engagement.

## 8 Limitations and future research

While this study provides significant insights into understanding how mindfulness affects creative engagement among employees in high-constraint environments, several limitations exist.

First, the cross-sectional design, while appropriate for initial model testing, prevents definitive causal inferences. Although grounded in theory, the temporal sequence of variables warrants verification. Future longitudinal or experimental designs (e.g., mindfulness intervention studies) are needed to establish causality.

Second, although the study identifies the chain mediating role of institutional pressure and career adaptability, this mediating path may not hold in all contexts. For example, the effect of career adaptability might be moderated by individual traits (e.g., proactive personality) or organizational environments (e.g., innovation climate).

Third, A critical limitation that warrants further discussion is our treatment of institutional pressures primarily as a hindering stressor. However, the challenge-hindrance framework (Lepine et al., 2005) reminds us that not all pressures are detrimental. While our study focused on their resource-depleting role (e.g., perceived constraints from censorship), certain forms of institutional pressures could act as “challenge stressors.” For instance, clear policy guidelines or high professional standards might provide constructive goals and necessary resource support, thereby triggering positive stress and motivating creative engagement. Future research should adopt more nuanced measurements to differentiate between these subtypes of institutional pressures (e.g., differentiating obstructive red tape from motivating guidance) and examine their potentially divergent effects on employee outcomes. This will help clarify the boundary conditions under which institutional pressures suppress versus stimulate creativity.

Thus, building on these limitations, future research could (1) use longitudinal designs and mixed methods to explore the dynamic relationships between variables; (2) delve into other mediating variables and the roles of individual and organizational moderators; (3) develop and utilize differentiated scales to measure the challenge and hindrance dimensions of institutional pressures; (4) adopt more precise measurement tools to analyze different impact mechanisms on creative engagement, thereby further clarifying the boundary conditions of the mediating paths.

## Data Availability

The raw data supporting the conclusions of this article will be made available by the authors, without undue reservation.
